# Early Lyme disease with spirochetemia - diagnosed by DNA sequencing

**DOI:** 10.1186/1756-0500-3-273

**Published:** 2010-11-01

**Authors:** Sin Hang Lee, Veronica S Vigliotti, Jessica S Vigliotti, William Jones, Jessie Williams, Jay Walshon

**Affiliations:** 1 Department of Pathology, Milford Hospital, 300 Seaside Avenue, Milford, 06460, USA; 2 Department of Emergency Medicine, Milford Hospital, 300 Seaside Avenue, Milford, 06460, USA

## Abstract

**Background:**

A sensitive and analytically specific nucleic acid amplification test (NAAT) is valuable in confirming the diagnosis of early Lyme disease at the stage of spirochetemia.

**Findings:**

Venous blood drawn from patients with clinical presentations of Lyme disease was tested for the standard 2-tier screen and Western Blot serology assay for Lyme disease, and also by a nested polymerase chain reaction (PCR) for *B. burgdorferi *sensu lato 16S ribosomal DNA. The PCR amplicon was sequenced for *B. burgdorferi *genomic DNA validation. A total of 130 patients visiting emergency room (ER) or Walk-in clinic (WALKIN), and 333 patients referred through the private physicians' offices were studied. While 5.4% of the ER/WALKIN patients showed DNA evidence of spirochetemia, none (0%) of the patients referred from private physicians' offices were DNA-positive. In contrast, while 8.4% of the patients referred from private physicians' offices were positive for the 2-tier Lyme serology assay, only 1.5% of the ER/WALKIN patients were positive for this antibody test. The 2-tier serology assay missed 85.7% of the cases of early Lyme disease with spirochetemia. The latter diagnosis was confirmed by DNA sequencing.

**Conclusion:**

Nested PCR followed by automated DNA sequencing is a valuable supplement to the standard 2-tier antibody assay in the diagnosis of early Lyme disease with spirochetemia. The best time to test for Lyme spirochetemia is when the patients living in the Lyme disease endemic areas develop unexplained symptoms or clinical manifestations that are consistent with Lyme disease early in the course of their illness.

## Background

Lyme disease is a tick-borne human infection which is an imperative differential diagnosis for internal medicine physicians offering primary care to ambulatory patients in the endemic counties of the United States. Hematogenous dissemination of the *Borrelia burgdorferi *spirochetes from the initial skin site of a tick bite is believed to cause secondary skin lesions and extracutaneous manifestations in Lyme disease [1]. Borrelia spirochetemia, when validated, provides reliable objective evidence for the diagnosis of early Lyme disease, based on which timely appropriate treatment is instituted to avoid tissue damage and to prevent the infection from going into chronic phase. However, *B. burgdorferi *spirochetemia is transient, and the culture techniques which require at least 9 mL of plasma sample and may take several weeks to recover [2] are not practical as a routine diagnostic tool. Pathogenic *Borrelia burgdorferi *cells are known to exist in non-dividing or slowly dividing forms which may not generate a visible positive growth in artificial media at all [3]. The diagnosis of early Lyme disease has been a challenging task for the primary contact physicians practicing in the endemic areas [4].

The polymerase chain reaction (PCR) technologies for the study of the most conserved genospecies-specific *Borrelia burgdorferi *sensu lato16S ribosomal RNA gene, or 16S rDNA, have been used in epidemiology research [5,6]. Using a pair of specific TEC1 and LD2 primers for PCR, the chances of non-specific amplification of 16S rDNA derived from spirochetes unrelated to Lyme disease are minimized [7]. However, little attempt has been made to transfer this procedure into clinical laboratory practice because the method is not robust enough for routine diagnostic applications. We have recently refined this research tool with a nested PCR technology for DNA detection, followed by automated direct DNA sequencing for validation of the genospecies-specific *B. burgdorferi *sensu lato 16S rDNA in patient body fluids to further augment the sensitivity and specificity of the procedure as a clinical laboratory test [8]. Since the base sequence of the PCR-amplified spirochete DNA in this procedure is routinely validated by online sequence alignment algorithms with the GenBank database with a 100% identities match with an exclusive unique sequence for the molecular diagnosis to be established, there are no false positive results due to molecular misidentification. The nested PCR technology has increased the sensitivity of the commonly used one-round PCR NAAT for Lyme spirochete DNA by 100-1000 fold [8]. This report summarizes our experience in using this routine clinical laboratory test for molecular diagnosis of *B. burgdorferi *spirochetemia in an endemic suburban town during a summer season.

## Methods

From May 1 to November 30, 2009, 463 paired samples of EDTA-anticoagulated venous blood and venous blood without additives from patients suspected of having Lyme disease were received by the Milford Hospital-affiliated Milford Medical Laboratory to be tested for Lyme disease.

Of these 463 pairs of blood samples, 130 were collected on the order of the physicians working in the hospital emergency room (ER) and walk-in clinic (WALKIN) because clinical manifestations of the patients were suggestive of Lyme disease with or without the history of a recent tick bite. Milford is a suburban town in Connecticut in which Lyme disease is endemic.

Milford Hospital is a community hospital. Its ER and WALKIN have about 40,000 patient visits a year. The local residents and practicing physicians are aware that Lyme borreliosis should always be a differential diagnosis during the months from spring to fall when a patient presents with a recent onset of fatigue, skin rash, fever, muscle aches, neck pain, joint pains or lymphadenopathy, without a clear etiology. These symptoms and signs which may vary from patient to patient are recognized as common clinical presentations in early Lyme disease in the United States [9].

The remaining 333 pairs of blood samples were from patients referred by their primary care private physicians in the area for possible Lyme disease.

The 130 ER/WALKIN patients had an age range between 14 and 84 years old with a median age of 42. In comparison, the 333 patients referred from the private physicians' offices had an age range between 11 and 89 with a median age of 51.

For every pair of the blood samples received, the plasma was separated from the EDTA-blood for nested PCR/DNA sequencing for the detection of *B. burgdorferi *16S rDNA, which was performed at the Milford Medical Laboratory, a clinical laboratory approved by the Department of Public Health, State of Connecticut, under the Clinical Laboratory Improvement Act of 1988 to perform high-complexity laboratory testing, including PCR and DNA sequencing for the molecular identification of *Borrelia burgdorferi*. The latter methodology was published elsewhere [8]. Briefly, a 100 μL aliquot of the patient plasma was mixed with 200 μL 0.7 M ammonium hydroxide in a 1.5 mL Eppendorf tube for DNA extraction. The mixture was heated at 95-98°C for 5 min with closed cap, followed by 10 min with open cap. After the tube was cooled to room temperature, 700 μL of 95% ethanol and 30 μL of 3 M sodium acetate were added to the mixture. The mixture was centrifuged at 13,000 rpm (~16,000 *g*) for 5 min and the supernatant discarded. The precipitate was re-suspended in 1 mL of cold 70% ethanol. Then the suspension was centrifuged at 13,000 rpm for 5 min. After all liquid was discarded, the pellet was air-dried and re-suspended in 100 μL TE buffer with heating at 95-98°C for 5 min. The heated suspension was finally centrifuged at 13,000 rpm for 5 min. One μL of the supernatant was used for primary PCR to be followed by nested PCR amplification without further purification, using a ready-to-use HiFi^® ^DNA polymerase LoTemp^® ^PCR mix (HiFi DNA Tech, LLC, Trumbull, CT) in a total volume of 25 μL. A trace of the primary PCR products without purification was transferred by a micro glass rod to another 25 μL LoTemp^® ^PCR mix containing a pair of heminested (nested) primers for nested PCR amplification.

The primary PCR primers used were nucleotides LD1 (5'-ATGCACACTTGGTGTTAACTA) and LD2 (5'-GACTTATCACCGGCAGTCTTA) [5]. The nested PCR primers were nucleotides TEC1 (5'-CTGGGGAGTATGCTCGCA AGA) [7] and LD2 [5]. The thermocycling steps were programmed to 30-cycles at 85°C for 30 seconds, 50°C for 30 seconds, and 65°C for 1 minute after an initial heating for 10 minutes at 85°C, with a final extension at 65°C for 10 minutes for both primary and nested PCR in a TC-412 Thermal Cycler (Techne Incorporated, Burlington, NJ). All positive nested PCR products showing a band of expected target size on gel electrophoresis were subjected to direct automated DNA sequencing, using TEC1 nucleotide as the sequencing primer.

The serum sample was submitted for Lyme disease antibody screen by the 2-tier immunoglobulin M (IgM) and immunoglobulin G (IgG) enzyme-linked immunosorbent assay (ELISA) and Western Blot for the detection of antibodies against sonicated whole-cell *B. burgdorferi *by Quest Diagnostics Incorporated, Wallingford, CT, a recognized commercial reference clinical laboratory, according to the CDC guidelines [10].

Publication of general analytical data extracted from hospital records with concealed patient identities was approved by the Milford Hospital Institutional Review Board.

## Results

**Figure 1:**
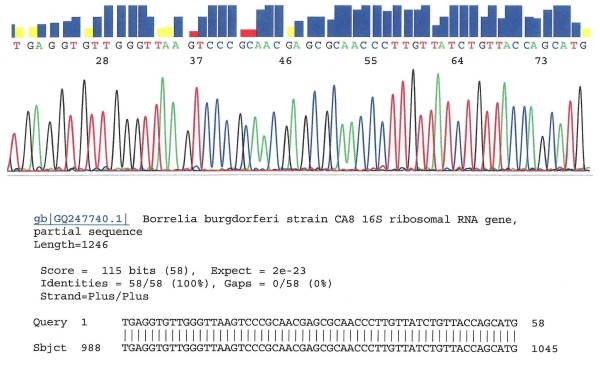
**DNA sequencing of *Borrelia burgdorferi *16S rDNA detected in the plasma of a spirochetemic patient**. This 58-base sequence was excised from an electropherogram generated by an ABI 3130 genetic analyzer. The template was the nested PCR amplicon generated by the TEC1 and LD2 primers. The sequencing primer was TEC1. BLAST alignment analysis validates the molecular diagnosis of hematogenous dissemination of Lyme disease in this patient. ABI, Applied Biosystems, Foster City, CA

As previously reported, nested PCR amplification of the conserved segment of *B. burgdorferi *sensu lato 16S rDNA for signature sequence analysis generated a 293 base-pair (bp) amplicon with the TEC1 and LD2 primers. After confirming a 100% identities match with a unique specific DNA sequence for *B. burgdorferi *sensu lato 16S rDNA stored in the GenBank database using the online Basic Local Alignment Search Tool (BLAST), the molecular identification of the nested PCR product as a genomic DNA of *B. burgdorferi *was established beyond a reasonable doubt. BLAST analysis of a 50-60 bp sequence downstream of the LD2 primer-binding site was more than adequate to achieve a very low E-value, which indicates that the chance of molecular misidentification is infinitesimal. A segment of the electropherogram containing the signature nucleotide sequence (Figure 1) was incorporated in the laboratory report for completion of an evidence-based molecular diagnosis of Lyme borrelia spirochetemia.

**Figure 2:**
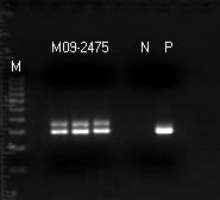
**Gel electrophoresis of nested PCR products of DNA from the plasma of a patient suspicious of Lyme disease (M09-2475)**. The sample was amplified by the TEC1 and LD2 primers and one major band had the molecular weight indistinguishable from the *B. burgdorferi *DNA control. P = *B. burgdorferi *16S rDNA nested PCR amplicon control; molecular size 293 base pairs. M09-2475 = Nested PCR products of questionable DNA isolated from a patient's plasma. The nested PCR was performed in triplicate to ensure technical accuracy. M = Molecular ruler. N = Negative control to rule out reagent contamination.

**Figure 3:**
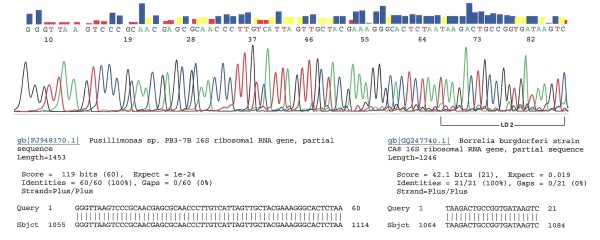
**DNA sequencing of the nested PCR products of case M09-2475, as illustrated in Figure 2**. The 21-base LD2 PCR primer-binding site for *B. burgdorferi *is marked on the right. A 60-base sequence on the left is validated to be that of a *Pusillimonas *16S rDNA based on GenBank database. This is a typical example of environmental bacterial 16S rDNA in patient's blood masquerading as *B. burgdorferi *16S rDNA.

**Figure 4:**
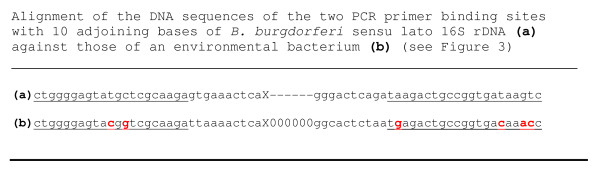
**Two partial DNA sequences retrieved from the National Center for Biotechnology Information database**. **(a) **GenBank Locus GQ247740, a 293-base long signature sequence for *B. burgdorferi *16S rDNA. TEC1 (left) and LD2 (right) PCR primer sites underlined. **(b) **GenBank Locus FJ948170, a 287-base long sequence of 16S rDNA for numerous environmental bacteria. TEC1 and LD2 primer sites underlined. Note 6 mismatched bases printed in red bold face. X------ = 231 bases in a sequence specific and unique for *B. burgdorferi *16S rDNA. X = 225 bases in a sequence nonspecific for environmental bacterial 16S rDNA. 000000 = 6 slots with no nucleotide bases. In the absence of a fully matched *B. burgdorferi *DNA, the PCR primers may bind to a partially matched non-target bacterial DNA templates which are not infrequently present in normal human blood. Only DNA sequencing can distinguish the 287 base-pair PCR amplicon of a common environmental bacterial 16S rDNA from a 293-base *B. burgdorferi *16S rDNA.

Our experience confirmed that PCR is not a specific tool for DNA identification, especially for the diagnosis of Lyme disease. From this series of 436 patients, 3 plasma samples were found to contain non-target DNA which led to generation of PCR products of a molecular size similar, but not identical, to that of the *B. burgdorferi *16S rDNA. These non-Lyme disease DNA molecules were amplified by the PCR primer pair designed for *B. burgdorferi *DNA replication. However, in the absence of a fully matched *B. burgdorferi *target DNA template, these unintended and non-target DNA molecules were amplified by the partially matched primers during the highly sensitive nested PCR process. One of such non-target PCR amplicons was only 6-bp shorter than the expected 293-bp *B. burgdorferi *16S rDNA fragment, as observed on gel electrophoresis (Figure 2). Only DNA sequencing could confirm that it was really a 287-bp 16S rDNA fragment of an environmental bacterium (Figure 3). As indicated in the GenBank database, the primer binding sites selected for PCR amplification of the most conserved 16S ribosomal RNA gene of the genospecies of *Borrelia burgdorferi *sensu lato also bear great similarities in DNA sequence with the 16S ribosomal RNA genes of other bacterial species (Figure 4).

There was an obvious difference in the test results between the 333 blood sample pairs from the patients referred to the laboratory by the individual private practitioners and the 130 blood ample pairs from the patients seen by the physicians at the ER and WALKIN. Of the blood samples from the former group of 333 patients, 28 (28/333), namely 8.4%, were found to be positive for the 2-tier IgM and IgG ELISA screen and further confirmed by Western Blot for the detection of antibodies against sonicated whole-cell *B. burgdorferi*. But all of the 333 companion plasma samples in this group were negative for *B. burgdorferi *nested PCR NAAT, indicating that there was no evidence of spirochetemia in these patients (Table 1).

**Table 1:** Comparison of nested PCR and 2-tier serology in detection of Lyme disease among 333 patients referred by private practitioners from offices

	Two-tier Serology		Total
		
	+	-	
Nested PCR +	0	0	0
Nested PCR -	28	305	333

Total	28	305	333

+ = positive

- = negative

Laboratory detection of Lyme disease among 333 patients referred from private offices:

Confirmed case prevalence = 28/333 = 8.4% (2-tier serology only)

Sensitivity of nested PCR = 0% (0/28)

Sensitivity of 2-tier seropositivity = 100% (28/28)

Of the blood sample pairs collected from the 130 patients visiting the ER and WALKIN, 2 (2/130), namely 1.5%, were found to be positive for the 2-tier Lyme disease serology test, and 7 (7/130), namely 5.4%, were found to contain *B. burgdorferi *16S rDNA. Of the 2 patients in this group, whose serum was positive for the 2-tier antibody test for Lyme disease, 1 was also found to have circulating *B. burgdorferi *DNA in the companion plasma. The other sero-positive patient did not have evidence of *B. burgdorferi *spirochetemia when the 2-tier Lyme disease antibody test became positive. In other words, among the 7 ER/WALKIN patients presenting with spirochetemia, 6 had *B. burgdorferi *DNA in their blood without the characteristic antibodies while 1 patient had both *B. burgdorferi *DNA and the characteristic Lyme disease antibodies in the blood (Table 2).

**Table 2:** Comparison of nested PCR and 2-tier serology in detection of Lyme disease among 130 patients visiting emergency room and walk-in clinic

	Two-tier Serology		Total
		
	+	-	
Nested PCR +	1	6	7
Nested PCR -	1	122	123

Total	2	128	130

+ = positive

- = negative

Laboratory detection of Lyme disease among 130 ER/walkin patients:

Confirmed case prevalence = (7+1)/130 = 8/130 = 6.2% (DNA sequencing or 2-tier serology)

Sensitivity of nested PCR = 87.5% (7/8)

Sensitivity of 2-tier seropositivity = 25% (2/8)

At the spirochetemic stage, 3 of the 7 patients had skin rashes. Two of the 3 skin lesions presented with a "bull's eye" appearance, considered typical of Lyme disease, and 1 was described as "hives". Most of the spirochetemic patients (5/7) stated that the duration of their chief complaint symptoms and signs lasted for about 24 hours before they decided to seek immediate medical attention. Two (2/7) of the patients had multiple joint pains or headaches for about 3 weeks before visiting the ER/WALKIN after noticing an additional chest pain or a skin rash. At the time of the initial visit, none of the spirochetemic patients registered a fever. On 4 patients for whom a CBC was ordered, 3 (3/4) showed slight leukocytosis with an increased percentage of neutrophils. One patient who had a concomitant chronic liver disease showed evidence of leukopenia. None of the 7 spirochetemic patients recalled a history of recent tick bites. As stated above, only one of the 7 spirochetemic patients (1/7) was found to be positive for the 2-tier serology test at the time of the initial visit. Follow-up information obtained from the primary care physicians of the patients confirmed that all presenting clinical symptoms and signs ascribed to Lyme borreliosis resolved completely after treatment with oral doxycycline, without recurrences in the ensuing 6-11 months. Only one of the 6 spirochetemic patients who were serologically negative at the initial visit was re-tested for possible rising antibody titers of Lyme disease, and the serology re-testing result was also negative. The major relevant clinical findings of the 7 spriochetemic patients were summarized in Table 3.

**Table 3:** Clinical summary of 7 early Lyme disease patients with spirochetemia

Age/Sex	Chief Complaint	Duration	Temp °F	CBC Results?	Hx Tick Bite?	Skin Lesion?	Serology	Follow up Serology
**1) 43/M**	Hives; Thoracic Spine Pain	24 hr	98.0	Not Done	NO	YES	ELISA = +, WB IgM = +	NONE

**2) 39/F**	Bilateral Leg Pain, Headache	24 hr	98.1	7.2 WBC; Elev Neut%	NO	NO	ELISA = - WB = -	NONE

**3) 15/F**	Shoulder Pain; Bilateral Leg Pain	24 hr	96.8	4.8 WBC; Elev Neut%	NO	NO	ELISA = -	ELISA = - 2 wks later

**4) 43/M**	Bull's eye rash	24 hr	98.3	Not Done	NO	YES	ELISA = -, WB = -	NONE

**5) 22/M**	Painful Inguinal Lymphadenopathy	24 hr	98.6	Not Done	NO	NO	ELISA = -, WB = -	NONE

**6) 52/M**	Multiple Joint Pain/Chest Pain	3 weeks/72 hr	97.7	10.8 WBC; Elev Neut%	NO	NO	ELISA = -	NONE

**7) 55/F**	Headache, Bull's eye rash	? 3 weeks	98.5	3.5 WBC; Decreased Neut%	NO	YES	ELISA = -	NONE

## Discussion

Accurate diagnosis of early Lyme disease plays a pivotal role in "curing" the infection with appropriate antibiotic treatment, and in preventing the infection from going into chronic phase which may cause debilitating tissue damage. However, the clinical manifestations of early Lyme disease are highly variable and often not easily distinguished from those caused by other illnesses. The commonly used 2-tier serology laboratory test which usually only turns positive during convalescence of the infection is reported to be negative or non-diagnostic in 75% of the "clinically confirmed" cases of early Lyme disease [4]. Testing for *B. burgdorferi *spirochetemia has been suggested to be the laboratory approach to diagnose early Lyme disease at the stage of hematogenous dissemination of the bacteria, which is believed to precede the appearance of the diagnostic antibodies [1,2,4]. However, the traditional microbiology blood culture techniques are not practical for the diagnosis of Lyme disease because it takes several weeks to recover a positive growth of the Lyme spirochetes in the liquid media. Attempts to culture *B. burgdorferi *spirochetes from patients' blood as a diagnostic tool have largely resulted in disappointments [11]. Non-dividing or slowly dividing *Borrelia burgdorferi *cells which do not generate a discernible positive culture in artificial liquid media are known to cause infections in animals [3]. The other alternative to detect this fastidious infectious agent in a patient's blood is to test for its genetic fingerprint materials, namely by a NAAT.

Several PCR-based nucleic acid amplification tests have been used for the detection of *B. burgdorferi *DNA in the blood samples of patients suffering from Lyme disease. However, their sensitivity is generally too low to be useful for clinical application [12-15] in part due to a lack of consistency of the *Borrelia burgdorferi *genetic materials targeted for PCR amplification by these methods. The lack of rigorous validation of the PCR products has also caused false positive results which can lead to inappropriate treatment with potentially serious complications [16,17]. Adoption of a NAAT procedure for the diagnosis of Lyme disease must proceed with caution.

Since all bacteria contain a 16S ribosomal RNA gene, or 16S rDNA, which differs from one another in their respective unique hypervariable regions, three oligonucleotide PCR primers, known as LD1, LD2 [5,6], and TEC1 [7], have been introduced to amplify a highly conserved region of the *B. burgdorferi *sensu lato 16S rDNA for its molecular fingerprint identification. In combination with the nested PCR and direct automated DNA sequencing technologies, these genospecies-specific PCR primers are useful in generating reliable materials for sequence alignment analysis using the online GenBank database as the standard for validation of the *B. burgdorferi *sensu lato 16S rDNA [8]. The potential value of their clinical application in confirmation of early Lyme disease spirochetemia has been demonstrated by the results presented in this report.

One potential pitfall in targeting a highly conserved bacterial16S rDNA of the genospecies of *B. burgdorferi *sensu lato for molecular diagnosis of Lyme borrelia spirochetemia is that some environmental bacterial 16S rDNA fragments, which may be present in normal human blood samples [18,19], can be amplified by the chosen PCR primers, especially when the nested PCR technology is employed to increase the detection sensitivity (Figures 2, 3, 4). This kind of potential false positive result generated by a non-specific PCR can be eliminated by routine direct DNA sequencing of all putative PCR-positive materials with their signature sequences validated through online GenBank sequence alignment algorithms (Figure 1).

In one residential suburb where Lyme disease is endemic, we found that 5.4% of the ER/WALKIN patients presenting with Lyme disease-like clinical manifestations were shown to have *B. burgdorferi *spirochetemia while none (0%) of the patients referred to the laboratory from their private doctors' offices with the same differential diagnosis had evidence of spirochetemia when tested by the same procedure. In comparison, only 1.5% of the ER/WALKIN patients in the same group were positive for the 2-tier antibody serology test for Lyme disease while 8.4% of the patients referred from the private doctors' offices were positive for the 2-tier serology test. These findings seem to indicate that the best time for detecting spirochetemia in early Lyme disease is when the onset of the clinical manifestations is noticed by the patient. Such immediate medical attention is probably only available at the ER or WALKIN in most endemic regions. Waiting for a scheduled appointment to the regular private doctor's office may miss the window of opportunity in DNA detection at the time when the Lyme disease bacteria are circulating in the blood, but only briefly.

In our series, 6 of the 7 (85.7%) PCR-detected, DNA sequencing-confirmed Lyme spirochetemic patients did not develop the 2-tier Lyme disease antibodies at the time of initial laboratory testing. Since these patients were all suspected of suffering from Lyme borreliosis based on clinical manifestations alone, they were prescribed a short course of preventive doxycycline while waiting for the laboratory test results. The antibiotics would be discontinued when the 2-tier serology screen test and the PCR test results were both found to be negative. All ER/WALKIN patients were referred back to their regular primary care physicians for follow up, and most private healthcare practitioners did not order additional serology tests for these patients. Therefore, it is not known if these 6 sero-negative, proven spirochetemic patients would turn sero-positive for the 2-tier serology test during their long-term convalescence. If no further follow-up serology tests were ordered, or if the subsequent 2-tier antibody tests turned out to be negative as a result of the initial partial treatment [20,21], these 6 Lyme disease patients would have been classified as having "no evidence of Lyme disease", except for the DNA evidence of Lyme spirochetemia. These clinical observations emphasize the importance of public education in the diagnosis of Lyme borrrelial spirochetemia. Early Lyme disease is essentially a patient-initiated laboratory diagnosis under the guidance of an alert physician. The patients generally control the window of opportunity for the detection of spirochetemia which is transient and brief. The time points of spirochetemia may vary from patient to patient.

## Conclusion

We found DNA evidence of *B. burgdorferi *spirochetemia in 7 of 130 (5.4%) ER/WALKIN patients with clinical manifestations of early Lyme disease. During the same period, we found no DNA evidence of spirochetemia in 333 patients who were referred from private physicians' offices for Lyme disease tests. In comparison, 28 of the 333 (8.7%) patients from the private physicians' offices were positive for the 2-tier Lyme disease antibody test whereas only 2 of the 130 (1.5%) ER/WALKIN patients were positive for the 2-tier serology test. Only 1 of the ER/WALKIN patients was positive both for the *B. burgdorferi *DNA and for the 2-tier antibody test at the same time. Based on these findings, we conclude that molecular testing for detection of *B. burgdorferi *spirochetemia should be a supplement to the standard 2-tier serology assay for all ER/WALKIN patients with clinical manifestations of early Lyme disease. Relying on a serology test alone may miss the diagnosis of 85.7% of the early Lyme disease, which can be confirmed by a blood NAAT for spirochetemia.

## Abbreviations

TEMP: temperature; CBC: complete blood count; WBC: white blood count; ELEV NEUT: elevated neutrophils; Hx: history; ELISA: Enzyme-linked immunosorbent assay; WB: Western Blot; +: positive; -: negative

## Competing interests

The authors declare that they have no competing interests.

## Authors' contributions

SHL conceived of the study, participated in its design and coordination and helped draft the manuscript. VSV, JSV and WJ participated in study conception, data acquisition, and laboratory data analyses. JW and JW participated in study conception, design, and clinical evaluation of patients. All authors read and approved the final manuscript.
